# Can Inhibitors of Snake Venom Phospholipases A_2_ Lead to New Insights into Anti-Inflammatory Therapy in Humans? A Theoretical Study

**DOI:** 10.3390/toxins9110341

**Published:** 2017-10-25

**Authors:** Thaís A. Sales, Silvana Marcussi, Elaine F. F. da Cunha, Kamil Kuca, Teodorico C. Ramalho

**Affiliations:** 1 Department of Chemistry, Federal University of Lavras, P.O. Box 3037, 37200-000 Lavras, MG, Brazil; thaissales194@hotmail.com (T.A.S.); marcussi@dqi.ufla.br (S.M.); Elaine_cunha@dqi.ufla.br (E.F.F.d.C.); teo@dqi.ufla.br (T.C.R.); 2 Biomedical Research Center, University Hospital Hradec Kralove, 500 05 Hradec Kralove, Czech Republic; 3 Center for Basic and Applied Research, Faculty of Informatics and Management, University of Hradec Kralove, Rokitanskeho 62, 500 03 Hradec Kralove, Czech Republic

**Keywords:** experimental model, *sv*PLA_2_, vanillic acid

## Abstract

Human phospholipase A_2_ (*h*PLA_2_) of the IIA group (HGIIA) catalyzes the hydrolysis of membrane phospholipids, producing arachidonic acid and originating potent inflammatory mediators. Therefore, molecules that can inhibit this enzyme are a source of potential anti-inflammatory drugs, with different action mechanisms of known anti-inflammatory agents. For the study and development of new anti-inflammatory drugs with this action mechanism, snake venom PLA_2_ (*sv*PLA_2_) can be employed, since the *sv*PLA_2_ has high similarity with the human PLA_2_ HGIIA. Despite the high similarity between these secretory PLA_2_s_,_ it is still not clear if these toxins can really be employed as an experimental model to predict the interactions that occur with the human PLA_2_ HGIIA and its inhibitors. Thus, the present study aims to compare and evaluate, by means of theoretical calculations, docking and molecular dynamics simulations, as well as experimental studies, the interactions of human PLA_2_ HGIIA and two *sv*PLA_2_s_,_ *Bothrops* toxin II and Crotoxin B (BthTX-II and CB, respectively). Our theoretical findings corroborate experimental data and point out that the human PLA_2_ HGIIA and *sv*PLA_2_ BthTX-II lead to similar interactions with the studied compounds. From our results, the *sv*PLA_2_ BthTX-II can be used as an experimental model for the development of anti-inflammatory drugs for therapy in humans.

## 1. Introduction

The inflammatory process involves a complex cascade of biochemical and cellular events, and it is an innate reaction of the organism that occurs in tissue in response to any cell injury from any dangerous agent: physical, chemical or biological. One of the stages of the inflammatory process is the breakdown of membrane phospholipids by phospholipases A_2_ (PLA_2_), which generates fatty acids, such as arachidonic acid (AA) and lysophospholipids. Oxidation of AA generates inflammatory mediators, such as prostaglandins, thromboxanes and leukotrienes, through the action of cyclooxygenase (COX) and lipoxygenase (LOX) enzymes. In addition to AA, the breakdown of membrane phospholipids generates lysophospholipids, a precursor of platelet-activating factor (PAF), another potent inflammatory mediator [1,2].

For the treatment of these inflammatory conditions, the Non-Steroidal Anti-Inflammatory Drugs (NSAIDs) are the most commonly employed drugs [3]. Their wide use throughout the world is due to the large number of diseases involving inflammatory disorders, the spread of rheumatic diseases, and an increase in the life expectancy of the population. Despite their widespread utilization, the prolonged use of this class of anti-inflammatory drugs causes several side effects, such as gastrointestinal toxicity and hepatotoxicity, among other diseases [4,5,6]. For this reason, there is great interest in the development of new compounds that can act as anti-inflammatory drugs, but with fewer side effects.

Despite their structural differences, all NSAIDs have a similar action mechanism, and are inhibitors of COX enzymes [7]. Recent studies have reported that the anti-inflammatory action of NSAIDs occurs by inhibition of the COX-2 isoform. However, the other products of the inflammatory cascade are also involved in inflammatory conditions, and the inhibition of the COX pathway may accentuate the LOX activity, and consequently increases leukotrienes production, the other product of arachidonic acid degradation [8,9,10]. In this way, the inhibition of the PLA_2_, which can act at the top of the cascade, is a promising alternative, since at the same time that it decreases the COX pathway, it also regulates the production of leukotrienes and the PAF-AH. Despite having great importance, only a few theoretical studies have been devoted to this topic and currently secreted PLA_2_ enzymes have not been explored as a molecular target by medicinal chemistry [11].

Among the various classes of existing PLA_2_, the human secreted PLA_2_ of the IIA group (HGIIA) belongs to the group of PLA_2_ that is the most associated with diseases and consequently are the target enzyme for inhibition [11]. Since human enzymes are difficult to obtain, some experimental models are generally employed for their study [12,13,14,15,16]. In this line, some enzymes that would possibly serve as an experimental model are the snake venoms PLA_2_ (*sv*PLA_2_). The secreted PLA_2_ from snake venoms are distributed in Subgroups I and II of the secreted PLA_2_ group_._ Of these, the crotalid and bothropic PLA_2_ are part of the II group, which is the same group as the HGIIA, which is a human PLA_2_. Crotoxin B (CB) (PDB ID 2QOG, UniProtKB AC: P24027) is the basic part of the Crotoxin (Cro), and its toxic part. Crotoxin was the first animal toxin to be purified and crystallized and is the main protein present in venom of the *Crotalus durissus terrificus* (South American rattlesnake) [17]. The *Bothrops* toxin II (BthTX-II) is another basic PLA_2_ isolated from *Bothrops jararacussu* venom. This myotoxic toxin is also known for its edematogenic and hemolytic effects and for its ability to induce platelet aggregation and secretion [18].

The use of *sv*PLA_2_ for understanding the activity and action mechanisms of the human PLA_2_ HGIIA has been proposed, as there is a high similarity between *sv*PLA_2_ and HGIIA PLA_2_ [19]. Moreover, the use of snake venom toxins could also be justified, because they are rich in Group I and II secreted PLA_2_, especially Elapidae and Viperidae families [20]. It should be kept in mind that despite this high similarity, it is not clear if these enzymes can perform similar interactions and if the *sv*PLA_2_ can really be employed as an experimental model to describe the HGIIA interactions, since some works reveal the contrary [21]. Thus, the objective of this work was to evaluate, experimentally, the phospholipasic activity of vanillic acid (VA) on *sv*PLA_2_ enzymes, such as BthTX-II and CB. In addition, we compare, theoretically, the interactions of these enzymes with the interaction of the same compound with HGIIA. Finally, two molecules rationally modified from the VA molecule were proposed to improve their interaction of the VA with HGIIA and to develop new potential anti-inflammatory drugs.

## 2. Results

### 2.1. Experimental Assays

**Figure 1:**
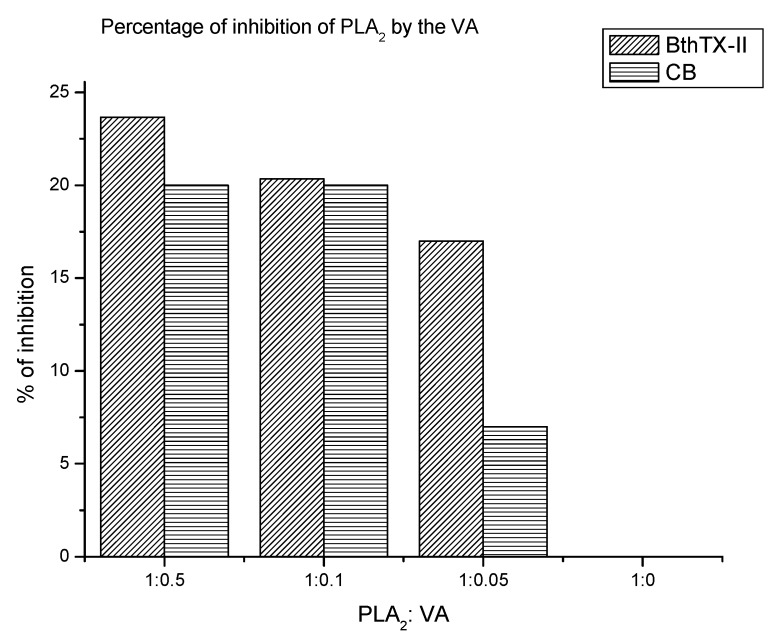
Percent inhibition of phospholipase A_2_ activity caused by vanillic acid (VA), for the phospholipases A_2_ isolated from snake venom *Bothrops* toxin II (BthTX-II) and Crotoxin B (CB).

Figure 1 contains the percent inhibition of both *sv*PLA_2_ by VA in relation to the different concentrations of this molecule. It is possible to observe that *sv*PLA_2_ BthTX-II presented a higher activity and value of inhibition percentage by the VA. In relation to the halo of activity (Figure S1 of the Support Material), the sample with the highest proportion of VA presents the lowest activity halo for both enzymes, which indicates that VA decreases the activity of both *sv*PLA_2_. In addition, the inhibition percentage values also indicate that vanillic acid is able to inhibit *sv*PLA_2_. Figure 1 shows that the highest proportion of VA tested was responsible for the highest percentage of inhibition for both enzymes, equivalent to 23.7% for the BthTX-II and 20% for CB.

### 2.2. Alignments of Amino Acid Sequences of svPLA_2_ and Human PLA_2_

Two alignments of primary amino acid sequences of BthTX-II (PDBID 3JR8), CB (PDBID 2QOG), and HGIIA (PDBID 3U8D) were performed and can be seen in Figures S2 and S3 of the support material. One is for calculating the percentages of identity and similarity between the enzymes, while the other is focusing on charge distributions and hydrophobicity of the three PLA_2_. The results of the first alignment showed 64.0% identity (84.4% similar) for alignment BthTX-II vs. CB (3-143:4-144); 53.1% identity (72.0% similar) for HGIIA vs. BthTX-II (3-145:3-143); and 55.2% identity (81.1% similar) for the alignment of the HGIIA vs. CB (3-145:4-144). For the second alignment, it is possible to observe that, although there are a few differences in some residues, the enzymes present groups (hydrophobic, negatively or positively charged residues) that behave similarly in the same positions, in most cases.

### 2.3. Theoretical Calculations

#### 2.3.1. Molecular Docking Calculations

**Figure 2:**
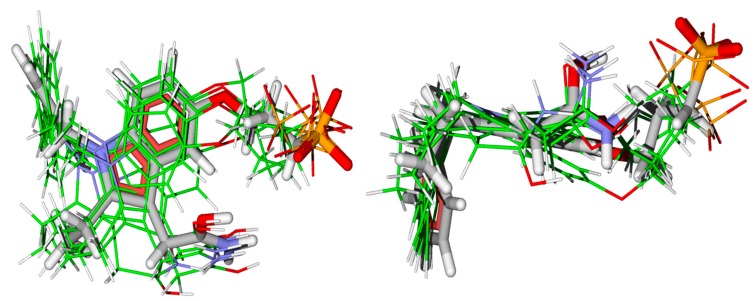
Superposition of the obtained poses with the active ligand U8D, obtained by re-docking calculation.

To validate the methodology, re-docking was performed on the human enzyme, under the same conditions, to compare the structure obtained in the theoretical calculation with the ligand structure of the U8D present in the crystal. The root-mean-square deviation (RMSD) obtained in re-docking was zero for all structures, which means that the structures obtained presented little alteration in relation to the average structure, which is satisfactory. The overlap of the obtained poses with the U8D active ligand are shown in Figure 2. As can be seen, there was no significant variation in the structures theoretically obtained with the active ligand structure of the 3U8D complex. Therefore, this result can validate our docking study.

**Figure 3:**
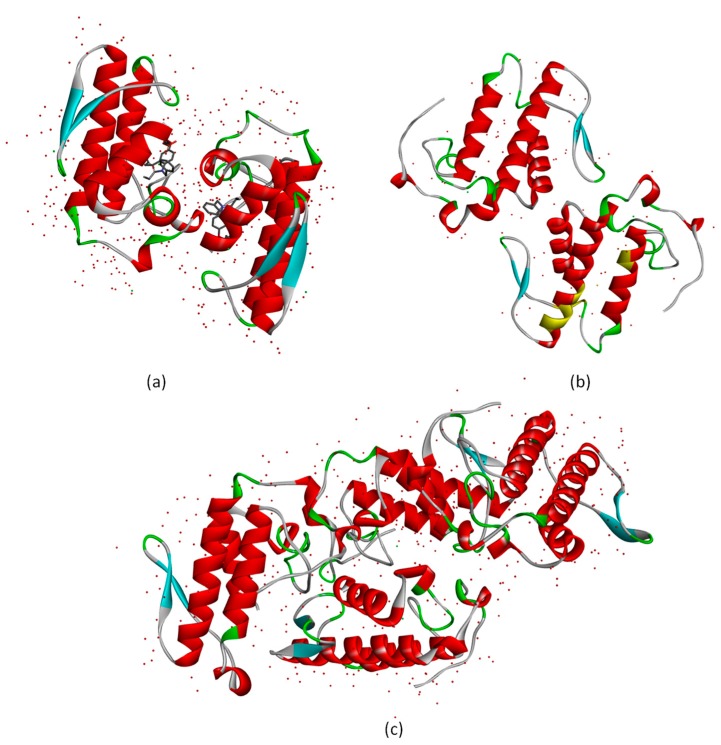
Three dimensional structures of secretory phospholipases A_2_: (**a**) represents the structures of HGIIA, with 3U8D PDB code; (**b**) represents the BthTX-II structure, with 3JR8 PBD code; and (**c**) is the 3D structure of CB, PDB code 2QOG.

Afterwards, the docking analysis of the vanillic acid with all enzymes was performed, and the results are reported in Table 1. As can be seen in Table 1, the interaction energies and the score function obtained for the enzyme HGIIA and BthTX-II were very close. This result does not apply to the third phospholipase CB. Both *sv*PLA_2_ have high similarity to the human enzyme HGIIA, however, only CB has four subunits composed of two equal dimmers, as seen in Figure 3. Each of these two dimmers is similar to the other PLA_2_ studied. BthTX-II and HGIIA enzymes have only two subunits in their active conformation, and this small structural difference can make the BthTX-II enzyme a little more appropriate to help describe the interactions that occur in the human enzyme.

**Figure 4:**
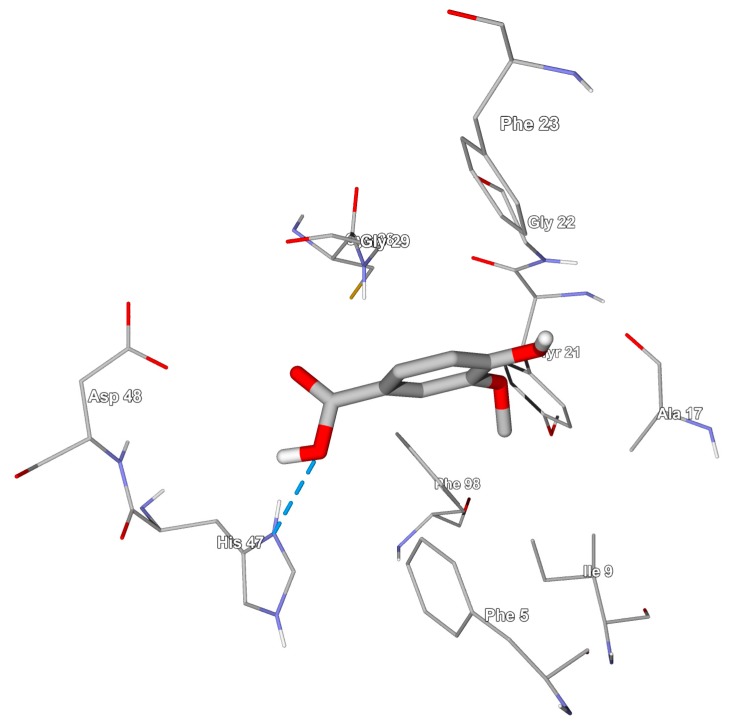
Hydrogen bond made between a vanillic acid molecule and the His 47 residue of PLA_2_ HGIIA, whose length is 2601 Å.

In relation to the hydrogen bond energies, the *sv*PLA_2_ had the lowest values, different from the HGIIA that have approximately −2.21 KJ mol^−1^. Despite of this difference in energies, just one hydrogen bond between the Histidine 47 residue of HGIIA and VA occurs, which can be seen in Figure 4. The bond length is 2.601 Å, and bonds longer than 2.5 Å are not very stable [22].

Through the superposition of the VA and the active ligand U8D, in Figure S4 of the support material, it is possible to deduce that VA leads to similar hydrophobic interactions. This means interactions between U8D and the hydrophobic residues of HGIIA, since the aromatic rings of VA are localized very close to the aromatic ring of the U8D. In addition, the oxygen atoms of the VA carboxyl group are also near the oxygen groups of the U8D molecule. Figure S5 shows the vanillic acid molecule inside the cavity of the HGIIA enzyme. As can be seen, VA occupies only a part of the cavity.

**Figure 5:**
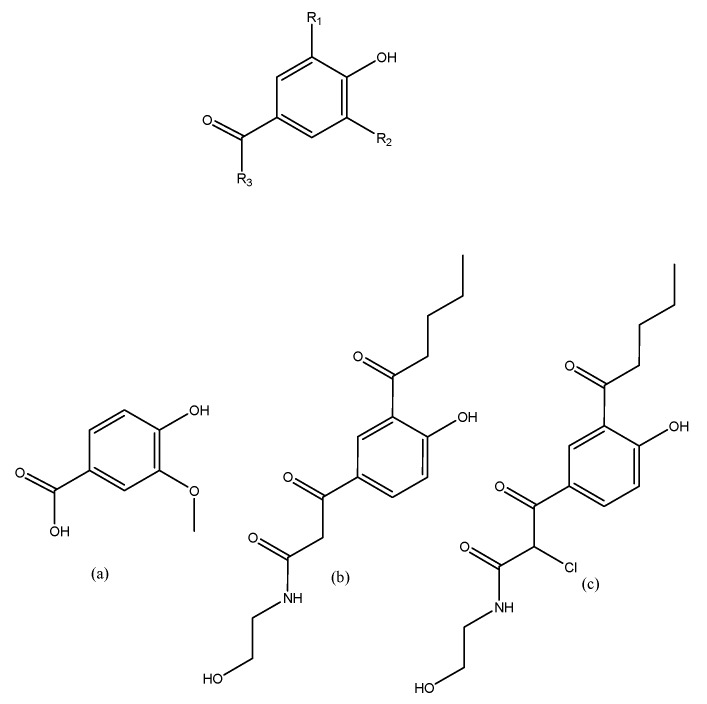
Modifications rationally proposed to improve the inhibitory activity of vanillic acid. On the top of the figure is the general structure; the molecule represented in (**a**) is the vanillic acid (VA), (**b**) is the first modification, named analogue I, and (**c**) is the second modification, named analogue II.

If new radical groups are rationally added in a vanillic acid molecule to take up all the cavity space, it is possible that the interaction of these compounds increases. Based on this idea, and considering the composition of the residues that are in the active site, two VA modified molecules were supposed, and docking analysis of their energies was performed. The proposed modifications are shown in Figure 5.

The docking calculation of the modified VA structures (analogues I and II) also were performed, and the results are displayed in Table 2. As can be seen, all results are better than the unmodified VA molecule (Table 1), which means that the modifications are satisfactory. The hydrogen bond energies have improved and are more similar between BthTX-II and HGIIA than the unmodified VA molecule. Moreover, the analogues followed the same interaction pattern, having more affinity for BthTX-II, followed by HGIIA and CB, the energies of the first two being very similar. The interaction energy between analogue I and the enzymes BthTX-II, HGIIA, and CB are −113.82, −107.12, and −71.93 KJ mol^−1^, respectively. For the interaction of these enzymes and analogue II, the interaction energies were −126.35, −115.38, and −55.64 KJ mol^−1^, respectively. These data also suggest that the BthTX-II serves as an experimental model to evaluate inhibitions in human secretory phospholipases of the IIA group.

#### 2.3.2. Molecular Dynamics Simulation

After the molecular docking study of VA in both PLA_2_, the structures obtained from the enzymes and the poses were analyzed by molecular dynamics. The root-mean square deviation (RMSD) and the number of hydrogen bonds were obtained for both systems. The first plot (Figure S6) shows the RMSD for each enzyme/inhibitor complex (HGIIA/VA; BthTX-II/VA, and CB/VA). In all systems, both VA and enzymes were stabilized, which indicates that all systems reached equilibrium. The *sv*PLA_2_ structures have more fluctuations over time, especially the CB/VA system.

For the HGIIA/VA system, which was the most stable, the equilibrium occurred as early as in the first picoseconds of simulation, and its maximum value was approximately 0.5 nm for the VA and 0.4 nm for the protein, both low values. This indicates that the VA ligand stabilized within the active site of the enzyme and that its interactions with HGIIA are favorable, proving its inhibitory potential. For the BthTX-II/VA system, the equilibrium was reached later for the ligand after 1000 ps, but it also occurred and was relatively maintained over time. Its maximum value was less than 1.2 nm while the RMSD of the BthTX-II enzyme did not reach 0.7 nm, which means that the permanence of the VA in the active site of the PLA_2_ BthTX-II is also favorable. The CB/VA complex provided the largest variation in position over time, but despite this, it also stabilized. The ligand varied widely in the active site of the CB enzyme, reaching a maximum RMSD of 2.5 nm for the ligand and 1 nm for the protein. Similar to the behavior adopted in the docking calculations, the BthTX-II enzyme was that which behaved more like the human enzyme HGIIA. This also suggests that BthTX-II is capable of aiding in the description of the experimental behavior of the human enzyme and that the CB PLA_2_ does not provide information of interactions between the VA ligand and the human PLA_2_.

**Figure 6:**
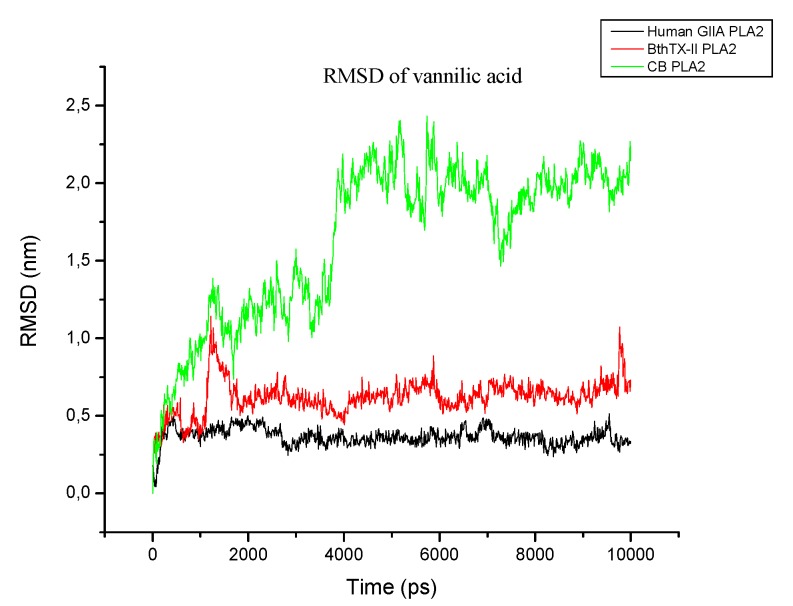
Comparison of root-mean square deviation (RMSD) of VA in each active site.

Comparing the RMSD between the enzymes, the HGIIA human PLA_2_ (*h*PLA_2_) was the most stable during the simulation, and BthTX-II was relatively stable. At the same time, the structure of CB *sv*PLA_2_ had many more fluctuations, as already mentioned. Turning now to the inhibitor in the three enzymes (Figure 6), it can be seen that the VA conformations in the enzymes HGIIA and BthTX-II have similar behaviors, unlike the ligand in the CB active site.

In relation to the hydrogen bonds carried out over time for the three studied PLA_2_ (Figure S7 of the support material), it is possible to observe that the *h*PLA_2_ HGIIA performed seven bonds during the molecular dynamics (MD) simulation, with approximately four being maintained most of the time. The BthTX-II *sv*PLA_2_, similarly to HGIIA, performed six hydrogen bonds in the MD simulation, four of which are more frequent over time. Regarding CB *sv*PLA_2_, unlike the other two phospholipases, CB PLA_2_ showed up to eight hydrogen bonds, but these were less stable, since they appear only at a few moments throughout the time.

## 3. Discussion

### 3.1. Can svPLA_2_/Inhibitors Describe the hPLA_2_/Inhibitors Interactions?

From the experimental data, it is possible to observe that the BthTX-II enzyme has more affinity with the VA molecule. This pattern was maintained in the docking and MD simulations, which indicate that the theoretical studies carried out are coherent with the experiment and suggest that HGIIA performs similar interactions between BthTX-II and VA. According to the literature, a degree identity over 35% is satisfactory [23]. Despite their similarity, it is important to comment that Kim and collaborators (2017) [21] found that the *sv*PLA_2_ purified from the venom of *Daboia russelli pulchella* (VRV-PL-VIII) is not appropriate as a model for describing the interactions between the human PLA_2_ and its inhibitors. As we can see in this work, the *sv*PLA_2_ CB, despite the high similarity with HGIIA, does not provide information about the interactions that occur between the HGIIA and VA, while the BthTX-II has a behavior similar to the human enzyme. This feature suggests that the structural similarity is a very important factor to consider, but is not the only factor. The other factor which plays an important role is the behavior of the enzyme in solution [21,24]. According to Kim and collaborators (2017) [21], the *sv*PLA_2_ does not provide any useful foundation for a prediction of the binding mode to specific ligands in a HGIIA complex. The authors conclude this based on the fact that the *sv*PLA_2_ enzymes have different behavior in solution, and because of this feature can interact with different chains (A or B) in a different mode. They found that the ligand FLSIK, in the HGIIA:FLSIK complex, does not interact with both chains, and as such, the chain B is not necessary for the inhibition activity, since the ligand interacts only with chain A. In other words, the authors found that the HGIIA acts as a monomer in solution. For the *sv*PLA_2_ that the authors chose (PLA_2_ purified from the venom of *Daboia russelli pulchella* (VRV-PL-VIIIA, svPLA_2_, UniProt accession code P59071, with 49% identity to HGIIA), the behavior in solution is different, and because of this, despite the high similarity, this *sv*PLA_2_ does not provide information of HGIIA interactions, as it acts as a monomer and *sv*PLA_2_ act as a dimmer.

**Figure 7:**
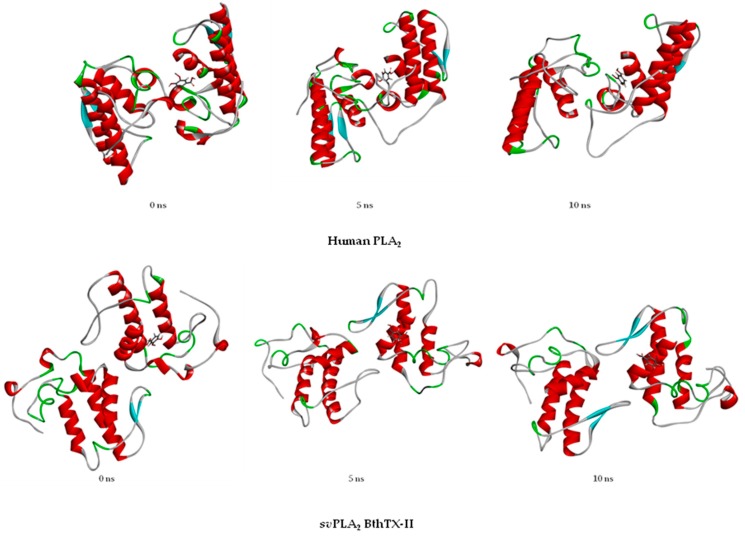
Comparison between the structure of the complex HGIIA/VA at the beginning (0 ns), middle (5 ns), and end (10 ns) of the molecular dynamics simulation, and comparison of the structures of the complex BthTX-II/VA at the beginning (0 ns), middle (5 ns), and end (10 ns) of the molecular dynamics simulation.

Similarly, for the authors, the simulations with HGIIA in the present work show that the ligand interacts with a single chain of the enzyme, which can be seen in Figure 7. The images represent the frames at the beginning, middle, and end of the simulation for the HGIIA/VA complex. As can be seen, the VA molecule is maintained in a single chain of the molecule at the three times. However, different from the conclusions of Kim and collaborators (2017) [21], in this work, we found that the *sv*PLA_2_ BthTX-II can provide a useful foundation for a prediction of the HGIIA binding mode. This fact is justified because the BthTX-II behavior in solution is similar to the HGIIA (Figure 7), different from the *sv*PLA_2_ CB tested in this work and the *sv*PLA_2_ tested by Kim and collaborators [21]. In Figure 7, the VA molecule also remains in the only chain of the enzyme most of time. As mentioned above, the CB PLA_2_, despite its high primary sequence similarity with HGIIA, acts as a tetramer, different from the other two tested PLA_2_. In addition, the PLA_2_ tested by Kim et al. (2017) [21], besides having less similarity to HGIIA, does not act as a monomer in solution.

Thus, for the similar interactions between HGIIA and BthTX-II, the similar behavior in solution, and for the high structural similarity of these compounds, it is possible that, experimentally, the vanillic acid acts in HGIIA in the same manner, with inhibition percentage values close to those of the BthTX-II results. Despite the differences in hydrogen bond energies in the docking calculations, the time dependent simulations show that the number of hydrogen bonds of BthTX-II and HGIIA are similar, and are maintained most of time, which also contributes to their similarity in interactions, contributing to the fact that the BthTX-II can be used as an experimental model for HGIIA.

**Figure 8:**
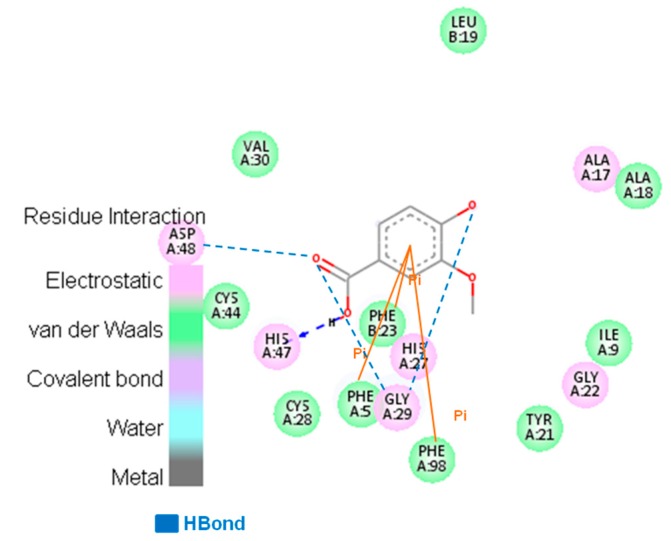
Pharmacophoric map of the interactions between HGIIA and vanillic acid (VA).

Moreover, the aromatic ring of the VA is in the same position as the active ligand of the 3U8D complex, suggesting that the same hydrophobic interaction can occur. With the structures obtained in the MD simulation, it was possible to create a pharmacophoric map of the HGIIA middle structures, which is approximately correspondent to the BthTX-II interactions. The maps are shown in Figure 8. The enzymes have similar hydrophobic interactions, and these interactions can explain the similar interaction energy obtained in molecular docking. In the map, it is possible to observe that the VA molecule performs π-π stacking interactions with phenylalanine residues and a hydrogen bond with glycine residues in both enzymes. The results obtained in this work are in agreement with the results obtained by Dileep et al. (2015) [25], who analyzed the effect of some phenolics on secretory PLA_2_ of the swine pancreas. The authors reported that vanillic acid interacts with this phospholipase by performing an H bond and by hydrophobic interactions with residues Phe 5, Leu 2, Phe 22, and Leu 31. Therefore, one secretory PLA_2_ that has more availability and is more easily obtained, which is BthTX-II, can be used as an experimental model for the study of mechanisms and the development of new inhibitors for the HGIIA PLA_2_ that are so important in regulations of the arachidonic acid pathway.

### 3.2. Searching Molecular Interactions of Vanillic Acid Analogs

**Figure 9:**
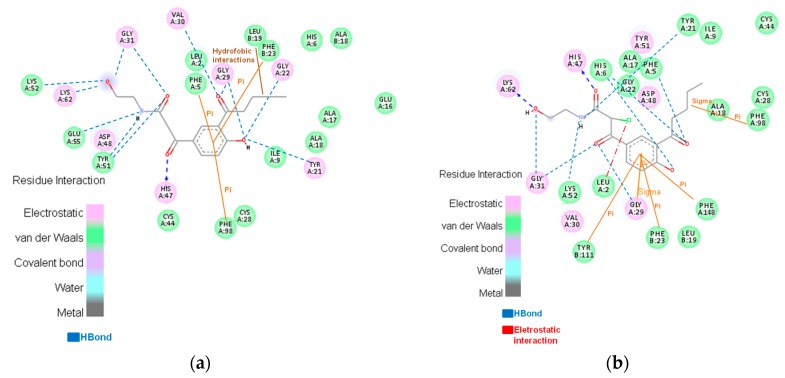
Interactions between the analogues I and II and HGIIA enzyme: (**a**) presents the interactions of the analogue I with HGIIA; (**b**) shows the interactions of the analogue II with the HGIIA enzyme.

With the modification of the VA molecule, the interactions increase significantly. As presented in Table 2, the interaction energies increase for both modifications with all PlA_2_. Moreover, the hydrogen bond energies for HGIIA and BthTX-II were very similar. Through the modifications of the VA molecule, the majority of the active site was occupied with radicals that interact with specific residues. This modification brings new hydrophobic interactions and hydrogen bonds, as can be seen in Figure 9, the pharmacophoric map. In addition, the chlorine atom in analogue II performs electrostatic interactions with HGIIA. With this, it is possible to conclude that vanillic acid can act as a base molecule for the rational development of new secreted PLA_2_ inhibitors. With better interaction, these new inhibitors can be more effective and selective for these enzymes, which enables the use of these molecules as possible anti-inflammatory drugs, with a different action mechanism from that of the current commercially available drugs.

## 4. Conclusions

In this work, a comparison of the HGIIA and *sv*PLA_2_ interactions was performed in order to clarify the discussion about the use of *sv*PLA_2_ as a model for analysis of human PLA_2_ interactions. In addition, two modified molecules from vanillic acid were theoretically proposed for increasing the inhibition of the VA molecule as well as its inhibitory effect. It is possible to conclude that the enzyme BthTX-II can provide useful information about the interactions of the potential inhibitors with HGIIA *h*PLA_2._ The other *sv*PLA_2_ tested, the Crotoxin B, or CB, does not present the same results, and so this enzyme cannot be used as an experimental model for HGIIA. It is also concluded that the primary sequence similarity is not the only factor to be considered, and the behavior of the enzyme in solution is an important factor for the comparison of interactions between the structurally similar enzymes.

This work is of great use, because we report a proof-of-principle study that snake venom toxins, more specifically *sv*PLA_2_, can be used as tools for studies in human PLA_2_, taking care in choosing the correct *sv*PLA_2_. Furthermore, it serves as evidence that both structural similarity and enzyme solution behavior are important to describe similarities in interactions of two or more enzymes. Lastly, vanillic acid has potential to inhibit secreted PLA_2_, and can be a base molecule for the development of molecules that can interact more strongly and can be more selective. The two rationally modified molecules developed from VA show better interaction energies than VA, which means that the developed molecules are more potent inhibitors than VA, and can be potential-use candidates for new anti-inflammatory drugs.

## 5. Materials and Methods

### 5.1. Experimental Assays

For the experimental analysis, the model of secretory *PLA_2_* employed was the *sv*PLA_2_ isolated from the species *Crotalus durissus terrificus* (CB) and *Bothrops jararacussu* (BthTX-II). The inhibition of phospholipase activity for vanillic acid was assessed using solid medium as described by Gutiérrez et al., 1988 [26], replacing agarose with agar and without the addition of erythrocytes. The substrate used was egg yolk. The egg yolk is a source of phospholipids, mainly phosphatidylcholine and phosphatidylethanolamine, thus forming an affordable and low-cost source for the detection of phospholipase activity [27]. The medium was prepared with 1% bacteriological agar, pH 7.2, and egg yolk diluted in phosphate-buffered saline (PBS) (1:3, *vv*^−1^). Also, 0.01 mol L^−1^ of CaCl_2_ and 0.005% of sodium azide was also added in the medium. After the gel solidified in plates, the treatments were applied in wells of approximately 0.5 cm of diameter. The two PLA_2_ isolated from snake venoms (BthTX-II and CB) were used to induce the breakdown of phospholipids. Each PLA_2_ and vanillic acid were diluted in CaCl_2_ solution and previously incubated in a water bath at 37 °C for 30 min, at the following ratios: 1:1, 1:0.5, 1:0.1, and 1:0.05 (PLA_2_/vanillic acid, *w/w*). The potential of vanillic acid in inhibiting PLA_2_ was evaluated after 18 h of incubation of the plates in a cell culture chamber at that same temperature. Controls containing only PLA_2_ were also evaluated. The formation of a clear halo around the well in the gel characterized the phospholipase activity, which was measured according to the halo diameter. The results were expressed as percentages of activity, and inhibition and the controls containing only venom were considered as having 100% phospholipase activity.

### 5.2. Alignments of Amino Acid Sequences

In order to verify the similarity of these enzymes with the human secretory PLA_2_ HGIIA, alignments were made using the LALIGN [28], a dynamic programming algorithm that determines similar regions of two protein sequences and other biomolecules. Additionally, the alignment of the UniProt [29] was employed to verify the presence of positive, negative, and hydrophobic residues. For the alignments, the primary sequences of these secretory PLA_2_ were downloaded from Expasy [30] in the categories of proteomics on the topic of protein sequences and identification, using the UniProtKB database [31]. In order to compare the interactions that occur between the ligands and all secretory PLA_2_, the same theoretical calculations were performed for both secretory PLA_2_.

### 5.3. Simulation Methods

#### 5.3.1. Docking Energies Calculations

To calculate the partial charges of ligands, the three-dimensional structures were previously created through the program PC Spartan^®^ (version Pro, Wavefunction, Inc., Ivine, CA, USA) [32], and the calculation was performed by the semi-empirical method AM1. After this, the ligands were docked inside the HGIIA (PDB code 3U8D, that have a resolution of 1.8 Å and are complexed with Ca^2+^, Cl^−^, and 3-{[3-(2-amino-2-oxoethyl)-1-benzyl-2-ethyl-1H-indol-5-yl]oxy}propyl)phosphonic acid (PDB code U8D)) using the software Molegro Virtual Docker (MVD^®^, version 2011.4.3.0, Qiagen Bioinformatics, Redwood City, CA, USA) [33]. The binding site was restricted into a sphere with a radius of 11 Å, and the residues within a radius of 8 Å were considered flexible. Fifty runs were performed, with 50 poses obtained for the analysis of the ligand-protein interactions and of the overlaps with the U8D inside of the human PLA_2_. The best conformation was selected, based on the best overlap and the interaction energy. For the analysis of the *sv*PLA_2_, the binding site, identified by the His47, was restricted into a sphere with 7 and 5 Å for the BthTX-II and CB, respectively, according to the size of the cavity. Since these enzyme structures do not have ligands, the best energy of interaction was taken into account. The selected conformations of all were used for the further MD simulation steps.

#### 5.3.2. Molecular Dynamics Simulations

Initial ligand configurations were produced using the Gaussian 09 Program [34] to construct the structures, and the Automated Topology Builder (ATB) server [35,36] to generate the topology and structure files. For the simulations, the force field used was GROMOS 96 54a7 [37], GROMACS program [38] (Version 5.1.2, Royal Institute of Technology and Uppsala University, Uppsala, Sweden). The enzyme/inhibitor complexes (HGIIA/VA, BthTX-II/VA and CB/VA) were constructed using the mentioned force field, in a volume simulation box of 645.57, 742.71, and 938.66 nm for each complex, respectively. For the energy minimization, the steepest descent algorithm was used, minimizing when the maximum force was <10.0 kJ/mol. After the minimization step, the complexes were submitted to molecular dynamics analysis for a time interval of 10 ns, and 1000 conformations were obtained for each complex. The equations of motion were integrated using the leapfrog scheme. The results were analyzed through the VMD^®^ program (version 1.9.2, University of Illinois at Urbana-Champaign, Champaign, IL, USA) [39] and Discovery Studio^®^ 3.5 (Accelrys, San Diego, CA, USA). The total energy, RMSD, and hydrogen bond graphs were generated to analyze the results through the Origin^®^ program (Version 3.5.0, Accelrys Software Inc., San Diego, CA, USA) [40,41,42,43].

## Figures and Tables

**Table 1:** Values obtained for the Score Binding Functions, Interaction Energy, and Hydrogen bonds for docking calculation of human phospholipase A_2_ (*h*PLA_2_) of the IIA group PLA_2_ (HGIIA), BthTX-II, and CB.

Enzyme	MolDock Score	Rerank Score	Interaction	HBond
*h*PLA_2_				
HGIIA	−69.38	−58.93	−75.35	−2.21
*sv*PLA_2_				
BthTX-II	−71.22	−57.02	−79.45	0.00
CB	−37.87	−35.17	−44.91	−0.02

**Table 2:** Values obtained for the Score, Interaction Energy, and Hydrogen Bond energies of the two analogues tested by docking calculation with the PLA_2_ HGIIA, BthTX-II, and CB.

Compound	Enzyme	MolDock Score	Rerank Score	Interaction	HBond
Analogue I	HGIIA	−101.52	−73.99	−107.12	−4.99
Analogue I	BthTX-II	−111.32	−69.64	−113.82	−4.61
Analogue I	CB	−72.26	24.57	−71.94	−5.14
Analogue II	HGIIA	−117.02	−26.35	−115.38	−4.63
Analogue II	BthTX-II	−123.33	−101.81	−126.35	−7.91
Analogue II	CB	−59.03	31.78	−55.64	−9.65

## Supplementary Materials

The following are available online at www.mdpi.com/2072-6651/9/11/341/s1, Figure S1: Halo of inhibition, in centimeters, formed by the inhibition of svPLA2svPLA2 isolated from BthTX-II and CB venom, by vanillic acid, Figure S2: Alignments of Human PLA2 HGIIA (3U8D) aminoacid sequences with the phospholipases A2 BthTX-II (3JR8) and PLA2 CB (2QOG), Figure S3: The amino acid sequence comparison between HGIIA, BthTX-II and PLA2 CB focusing on similar distribution of charged amino acid and hydrophobicity, Figure S4; Overlap of the active ligand of the 3U8D complex, of the enzyme HGIIA, with the vanillic acid obtained by the molecular docking, Figure S5: Volume of the cavity of the enzyme HGIIA with the molecule of vanillic acid anchored, Figure S6: Root Mean square deviation (RMSD) for the HGIIA/VA, BthTX-II/VA and CB/VA complexes, Figure S7: Hydrogen bonds carried out between vanillic acid and PLA2 enzymes.
