# Supplementary material for: Can Inhibitors of Snake Venom Phospholipases A2 Lead to New Insights into Anti-Inflammatory Therapy in Humans? A Theoretical Study
Source: Toxins (Basel). 2017 Oct 25;9(11):341. doi: 10.3390/toxins9110341 (PMC5705956; doi:10.3390/toxins9110341)
Supplement: Supplementary file 1 [file toxins-09-00341-s001.pdf]

Supplementary Materials: Can Inhibitors of Snake Venom Phospholipases A2 Lead to New Insights into Anti-Inflammatory Therapy in Humans? A Theoretical Study

Thaís A. Sales, Silvana Marcussi, Elaine F. F. da Cunha, Kamil Kuca and Teodorico C. Ramalho

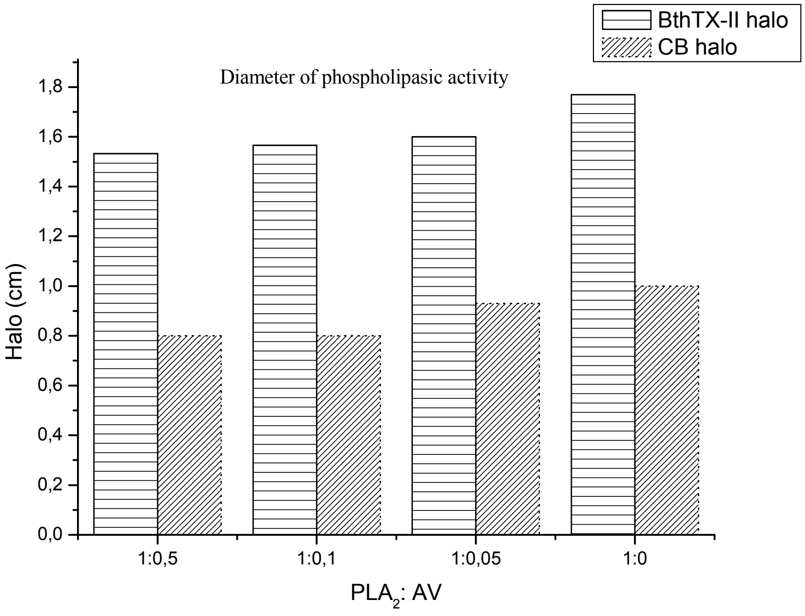

Figure S1. Halo of inhibition, in centimeters, formed by the inhibition of svPLA2svPLA2 isolated from BthTX-II and CB venom, by vanillic acid.

```
               10        20        30        40        50        60
3JR8    APDBIDCHAINSEQCENCEDLWQFGQMILKETGKLPFPYYTTYGCYCGWGGQGQPKDATD
        ::::::::::::::::::::.: ::..::  :: :   :.:. :::::::::::.::::::
2QOG    APDBIDCHAINSEQCENCESLLQFNKMIKFETRKNAVPFYAFYGCYCGWGGQGRPKDATD
              10        20        30        40        50        60

               70        80        90       100       110       120
3JR8    RCCFVHDCCYGKLTNCKPKTDRYSYSRENGVIICGEGTPCEKQICECDKAAAVCFRENLR
        :::::::::::::..:. : : : :: ..: : ::.:: :..::::::..:: :.:..:
2QOG    RCCFVHDCCYGKLAKCNTKWDIYRYSLKSGYITCGKGTWCKEQICECDRVAAECLRRSLS
              70        80        90       100       110       120

              130       140
3JR8    TYKKRYMAYPDVLCKKPAEKC
        :::..:: :::  :..:.: :
2QOG    TYKNEYMFYPDSRCREPSETC
             130       140
```

(**a**)

```
               10        20        30        40        50        60
3U8D    APDBIDCHAINSEQCENCENLVNFHRMIKLTTGKEAALSYGFYGCHCGVGGRGSPKDATD
        ::::::::::::::::::::.: .: .::    :::     :  :::.:: ::.:.:::::
3JR8    APDBIDCHAINSEQCENCEDLWQFGQMILKETGKLPFPYYTTYGCYCGWGGQGQPKDATD
               10        20        30        40        50        60

               70        80        90       100       110       120
3U8D    RCCVTHDCCYKRLEKRGCGTKFLSYKFSNSGSRITCAKQDSCRSQLCECDKAAATCFARN
        ::: .::::: .: .  :  :   :..:  .. : :..   :..:.::::::::.:: .:
3JR8    RCCFVHDCCYGKLTN--CKPKTDRYSYSRENGVIICGEGTPCEKQICECDKAAAVCFREN
               70          80        90       100       110       120

              130       140
3U8D    KTTYNKKYQYYSNKHCRGSTPRC
          ::.:.:. : .  :.  . .:
3JR8    LRTYKKRYMAYPDVLCKKPAEKC
                130       140
```

(**b**)

```
               10        20        30        40        50        60
3U8D    APDBIDCHAINSEQCENCENLVNFHRMIKLTTGKEAALSYGFYGCHCGVGGRGSPKDATD
        ::::::::::::::::::::.:..:..:::. : :.:.  :.::::.:: ::.: ::::::
2QOG    APDBIDCHAINSEQCENCESLLQFNKMIKFETRKNAVPFYAFYGCYCGWGGQGRPKDATD
              10        20        30        40        50        60

               70        80        90       100       110       120
3U8D    RCCVTHDCCYKRLEKRGCGTKFLSYKFSNSGSRITCAKQDSCRSQLCECDKAAATCFARN
        ::: .::::: .: :  :.::.  :..: ... :::.:    :. :.:::::..:: :. :.
2QOG    RCCFVHDCCYGKLAK--CNTKWDIYRYSLKSGYITCGKGTWCKEQICECDRVAAECLRRS
              70          80        90       100       110       120

              130       140
3U8D    KTTYNKKYQYYSNKHCRGSTPRC
         .::...:..: ...::  .  :
2QOG    LSTYKNEYMFYPDSRCREPSETC
               130       140
```

(**c**)

Figure S2. Alignments of Human PLA2 HGIIA (3U8D) aminoacid sequences with the phospholipases A2 BthTX-II (3JR8) and PLA2 CB (2QOG): (**a**) is the *sv*PLA2 aminoacids sequences alignment, (**b**) it's the alignment of the PLA_2 primary sequences HGIIA and BthTX-II and (**c**) represents the alignment performed with HGIIA and CB.

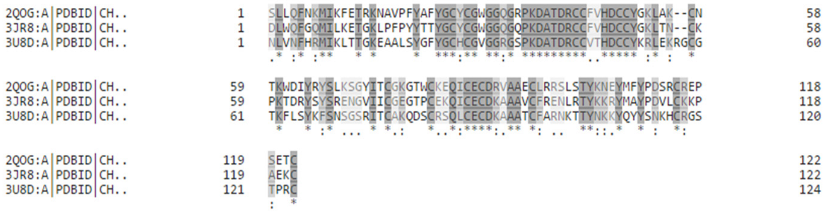

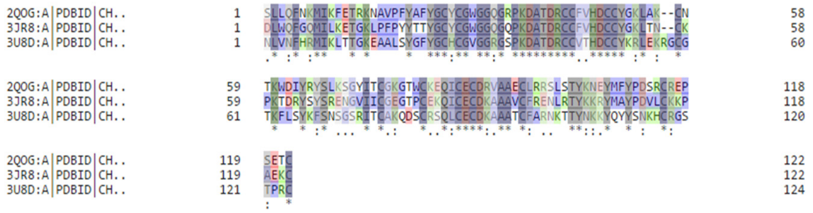

Figure S3. The amino acid sequence comparison between HGIIA, BthTX-II and PLA2 CB focusing on similar distribution of charged amino acid and hydrophobicity. The gray color represents the similarity between the amino acid sequences. The residues in purple represent the hydrophobic parts, in pink represent the negative parts and residues in green correspond to positive charged residues.

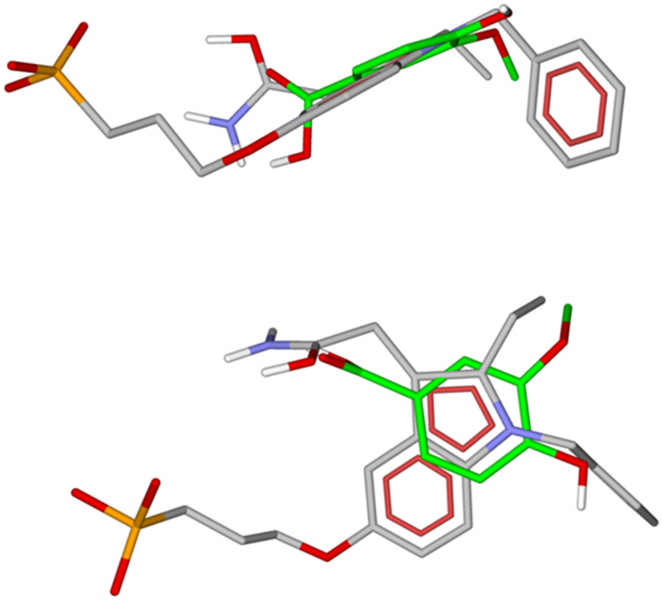

Figure S4. Overlap of the active ligand of the 3U8D complex, of the enzyme HGIIA, with the vanillic acid obtained by the molecular docking.

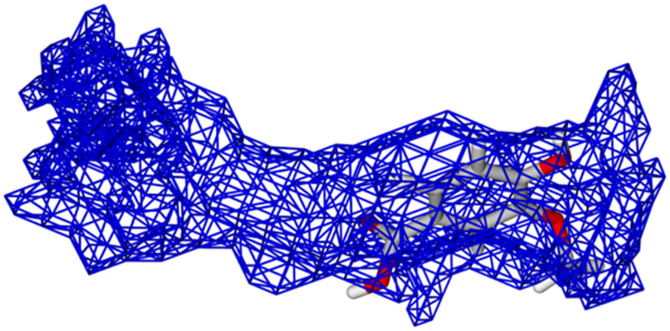

Figure S5. Volume of the cavity of the enzyme HGIIA with the molecule of vanillic acid anchored.

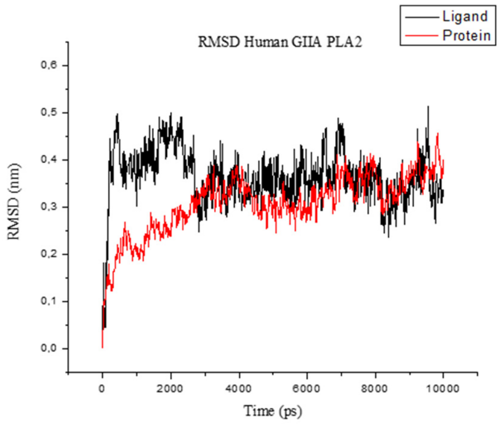

(**a**)

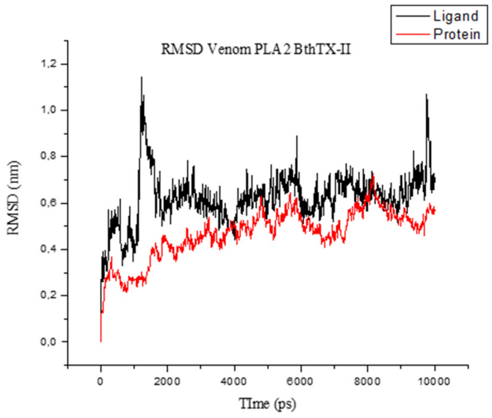

(**b**)

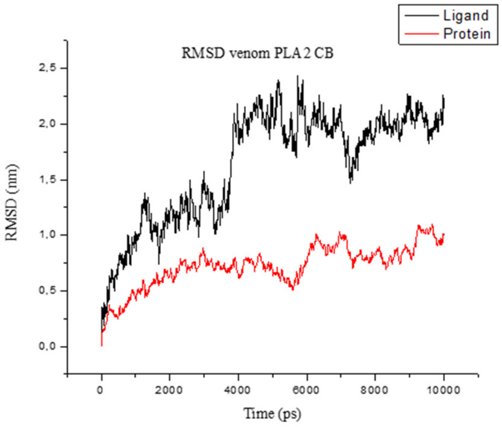

(**c**)

Figure S6. Root Mean square deviation (RMSD) for the HGIIA/VA, BthTX-II/VA and CB/VA complexes: (**a**) is the plot of RMSD for the HGIIA enzyme from the MD simulation, (**b**) is the RMSD for the BthTX-II toxin and (**c**) is the RMSD for the second toxin CB from the MD analysis.

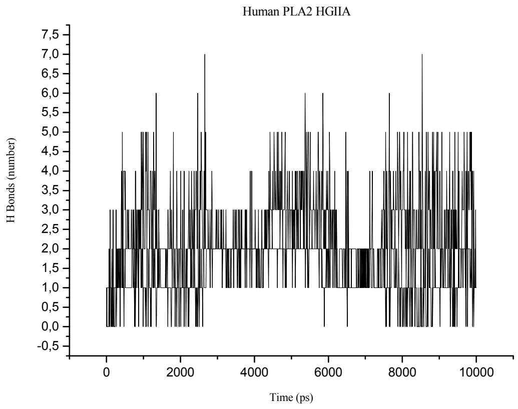

(**a**)

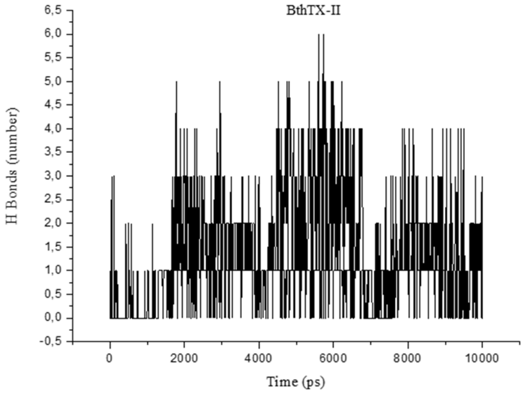

(**b**)

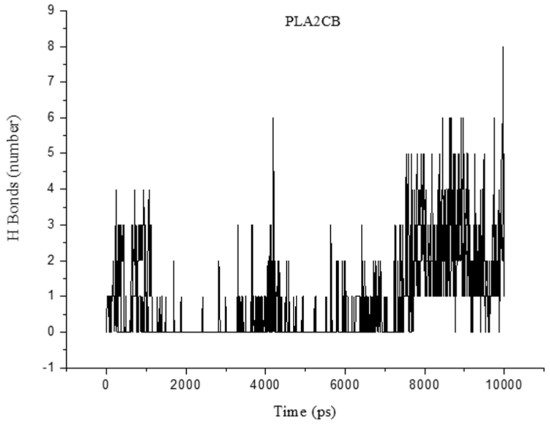

(**c**)

Figure S7. Hydrogen bonds carried out between vanillic acid and PLA2 enzymes. The first plot (**a**) is the Hydrogen bonds made with HGIIA, (**b**) is the Hydrogen bonds made with BthTX-II enzyme and (**c**) is the CB hydrogen bonds.
